# Using Fumed Silica to Develop Thermal Insulation Cement for Medium–Low Temperature Geothermal Wells

**DOI:** 10.3390/ma15145087

**Published:** 2022-07-21

**Authors:** Lan Shen, Huijing Tan, You Ye, Wei He

**Affiliations:** 1 State Key Laboratory of Oil and Gas Reservoir Geology and Exploitation, Chengdu University of Technology, Chengdu 610059, China; shenlan1807@163.com (L.S.); yeyou@stu.cdut.edu.cn (Y.Y.); 2021020363@stu.cdut.edu.cn (W.H.); 2 Key Laboratory of Deep Geodrilling Technology, Ministry of Natural Resources, China University of Geosciences (Beijing), Beijing 100083, China

**Keywords:** thermal insulation cement, vitrified microbubbles, fumed silica, geothermal well cementing, orthogonal test

## Abstract

During geothermal energy development, the bottom high-temperature fluid continuously exchanges heat with the upper low-temperature wellbore and the stratum during its rising process. Thermal insulation cement (TIC) can increase the outlet temperature, thus effectively reducing the heat loss of the geothermal fluid and improving energy efficiency. In this study, vitrified microbubbles (VMB) were screened out by conducting an orthogonal test of compressive strength (CS) and thermal conductivity (TC) on three inorganic thermal insulation materials (VMB, expanded perlite (EP), and fly-ash cenosphere (FAC)). Fumed silica (FS) was introduced into the cement with VMBs, as its significant decreasing effect on the TC. Moreover, a cement reinforcing agent (RA) and calcium hydroxide [CH] were added to further improve the CS of TIC at 90 °C. The fresh properties, CS, TC, hydration products, pore-size distribution, and the microstructure of the cement were investigated. As a result, a TIC with a TC of 0.1905 W/(m·K) and CS of 5.85 MPa was developed. The main conclusions are as follows: (1) Increasing the mass fraction of the thermal insulation material (TIM) is an effective method to reduce TC. (2) The CH content was reduced, but the C–S–H gel increased as FS content increased due to the pozzolanic reaction of the FS. (3) As the C–S–H gel is the main product of both the hydration and pozzolanic reactions, the matrix of the cement containing 60% FS and VMBs was mainly composed of gel. (4) The 10% RA improved the cement fluidity and increased the CS of TIC from 3.5 MPa to 5.85 MPa by promoting hydration.

## 1. Introduction

**Figure 1:**
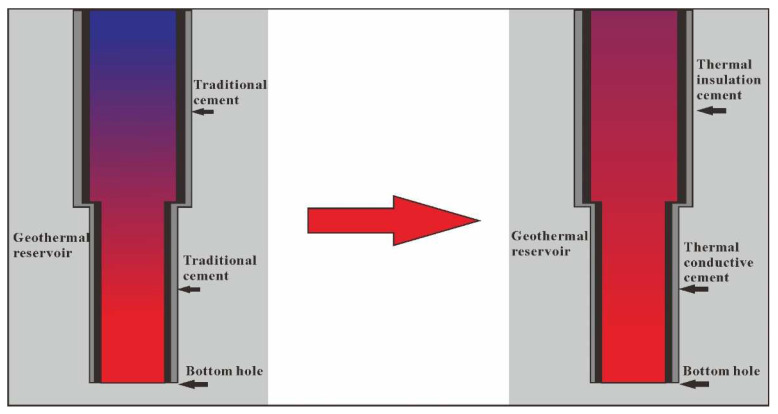
Diagram illustrating thermal-insulation cementing in the low-temperature zone of a geothermal well.

Geothermal energy is a clean and pollution-free renewable natural resource with large reserves [1]. The geothermal energy of China mainly consists of medium–low-temperature resources, and its development and utilization are currently mainly focused on hydrothermal resources [2,3]. The outlet temperature directly affects the economic evaluation of geothermal energy exploitation [4]. As shown in Figure 1, when geothermal fluid rises in the wellbore, it continuously exchanges energy with the wellbore and low-temperature formation, resulting in a decrease in the outlet temperature. A lower wellhead temperature is unfavorable for geothermal energy applications [3]. Well cementing is usually necessary during geothermal well construction, and the cement sheath occupies a large part of the wellbore cross-section. Using thermal insulation cement (TIC) to replace conventional cement could reduce the heat loss of the geothermal fluid, thereby increasing the outlet temperature and thermal recovery efficiency [3,5,6,7].

Single-well circulation systems for geothermal energy transfer have been proposed for many years [8]. However, the current main mining method is still a multi-well system, as the heat-exchange area of single-well circulation systems is small [9]. In production wells, the temperature of the bottom hole fluid is highest and decreases with increasing distance from the bottom hole, especially in the low-temperature well section near the ground [10]. Therefore, the application of TIC is necessary for geothermal production wells. Previous studies on modifying the thermal conductivity of cement have mainly focused on enhancing heat conduction in geothermal single-well heat-extraction technology [11]. Relatively few studies have been conducted on TIC for geothermal wells. The theory that decreasing the thermal conductivity of cement can significantly reduce heat loss was first proposed by Ichim et al. [12] in 2016; they later estimated the thermal properties of cement using a three-phase model for application in geothermal wells in 2018 [5]. Sugama and Tatiana assessed the possibility of using polymethyl hydrosiloxane-treated fly-ash cenospheres (FACs) to formulate a thermally insulated and thermal shock-resistant cementitious blend with calcium aluminate cement [13]. Zhang and Li developed a TIC for geothermal wells by introducing floating beads and expanded perlite (EP) into Class G oil-well cement [4]. Sliwa and Ciepielowska developed a TIC with a compressive strength (CS) of 8.841 MPa and a thermal conductivity of 0.566 W/m∙K by introducing rubber powder and glass microspheres into Portland cement [14]. However, to ensure a certain strength, the thermal conductivities of the developed materials are >0.5 W/(m∙K), which are not low enough.

Mixing thermally insulated materials (TIMs) into cement is a simple and effective method for improving thermal insulation performance. Currently, research on thermally insulated composite materials in the construction industry is relatively mature [15,16,17,18]. However, the environment of geothermal cementing is different from that of buildings. (1) High CSs are not required for TIC used in building external walls. The CS of the most developed TIC for buildings is less than 2.0 MPa, or even less than 0.3 MPa [19,20]. However, the CS of geothermal cement is generally required to be greater than 6.9 MPa for 24 h [21,22]. (2) Building materials must be fire resistant; the fire resistance of building materials is generally tested in dry, high-temperature conditions (above 600 °C) [23,24]. However, geothermally insulated cement for medium–low-temperature geothermal wells should be applied in a hydrothermal environment with an average temperature of 90 °C. In general, geothermally insulated cement should have not only low thermal conductivity but also maintain sufficient strength in a hydrothermal environment at ≈90 °C.

In previous studies, TIMs incorporated into cement were mainly divided into organic and inorganic materials. These conventional TIMs are composed of a wide network of solid material with air inclusions (pores) that are small enough to suppress convection within [25]. Organic TIMs usually consist of polystyrene particles, polyurethane particles, etc., while inorganic TIMs include FAC, EP, mineral wool, vermiculite, and so on [26]. The thermal decomposition of organic materials at high temperatures leads to poor durability. Thus, they are unsuitable for geothermal cementing [27]. Although inorganic materials usually have less of an effect on reducing the TC of cement, they are usually cheaper and have better durability [28,29]. Supplementary cementitious materials, including industrial by-products or natural pozzolans, are widely used as a partial replacement for cement to enhance mechanical and durability properties [30,31]. Recently, fumed silica (FS)-based insulation materials have attracted considerable attention, as they could largely increase the early strength and significantly decrease the TC of cementitious material [32]. However, few studies have been conducted on the application of FS in geothermal insulation cementing.

In this study, three inorganic TIMs (vitrified micro-bubbles (VMBs), EP, and FAC) were screened by conducting orthogonal tests of CS and TC. Second, FS was introduced into the cement with the optimized TIM. Third, the CS of the optimized cement formula was further improved by adding either a cement-reinforcing agent or calcium hydroxide [CH]. Finally, the effects of the TIMs on the fresh properties, CS, TC, hydration products, pore-size distribution, and microstructure of the cement were investigated. Table 1 provides a list of symbols and mathematical notations.

## 2. Materials and Methods

### 2.1. Materials

Class G oil-well cement was supplied by Jiahua Special Cement Co., Ltd., Leshan, China. The main components of the cement were C_3_S (54.2 wt%) and 2C_3_A + C_4_AF (18.4 wt%). The chemical and physical properties of the cement are listed in Table 2. The EP, VMBs, and FACs were purchased from Rongchangsheng Environmental Protection Materials Co., Ltd., Zhengzhou, China. The cement reinforcing agent No. SR (SR-RA) powder, with a silica ratio of 65.88%, pH of 9.53, and fineness modulus of 1.95%, was purchased from Enjoyable Ecology Engineering Technology Co., Ltd., Huaian, China; its specific properties are listed in Table 3. The FS, mainly composed of silicon dioxide (≥99.0 wt%), was supplied by Huofeng Thermal Insulation Material Co., Ltd., Langfang, China; its physical properties are listed in Table 4. The oxide compositions, as determined by X-ray fluorescence (XRF; PANalytical Axios) analysis of EP, VMB, FAC, and SR-RA, are listed in Table 5. Calcium hydroxide (CH) was purchased from Tianjin Hengxing Chemical Preparation Co., Ltd., Tianjin, China.

### 2.2. Mix Design and Sample Preparation

#### 2.2.1. Orthogonal Experiment Design

The orthogonal test method was used for multi-factor and multi-level research to determine the optimal scheme and significance of the factors [33,34]. In this study, the curing and testing temperatures (factor A), type of insulation material (factor B), and mass fraction of the insulation materials (factor C) were considered as the three major mix-design parameters. The three curing temperatures were 25, 60, and 90 °C, the three types of insulation materials were EP, VMB, and FAC, and the three TIM percentages were 10, 20, and 30% by weight. These factors and their corresponding levels are listed in Table 6. The L9 orthogonal array-full factorial experimental design of Taguchi in this study is presented in Table 7. The fourth column is blank and can be used for variance analysis as an error term [35,36]. Furthermore, the mix proportions of cement slurries for the orthogonal test are listed in Table 8.

#### 2.2.2. Preparation Procedure

**Figure 2:**
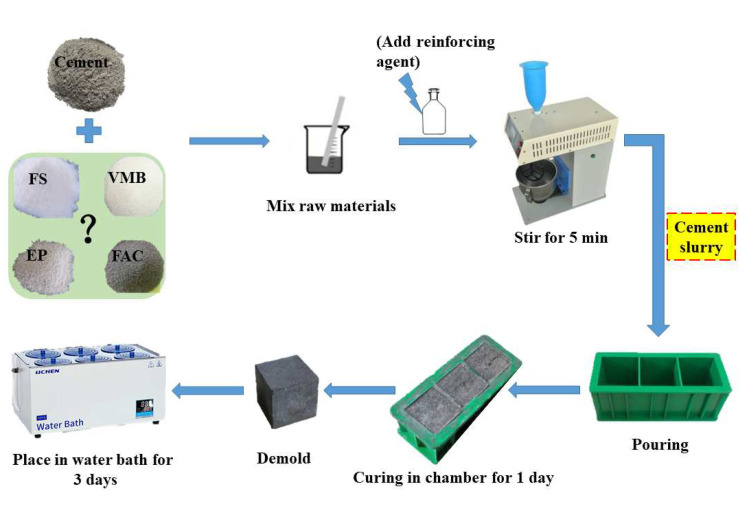
Preparation process of cement slurry and hardened samples.

The water-to-solid (W/S) ratio of cement slurries for the orthogonal test was maintained at 0.50 in order to eliminate the interference of water content. Class G oil well cement slurry (control) with a water-to-cement (W/C) ratio of 0.5 was prepared for comparison. Then, the W/S ratio of the slurries for the two-factor complementary test was adjusted to ensure that the cement slurries could be uniform. All of the dry raw materials were proportionally mixed and poured into water. An automatic planetary cement-paste blender (FY-681, Beijing Zhongke Jianyi Electronic Technology Co., Ltd., Beijing, China) was used to prepare the cement slurries according to the Chinese standard, GB/T 19139-2012 [37]. The stirring time was appropriately extended to ensure uniformity of the cement slurry. The mixed cement slurries were poured into 50 mm × 50 mm × 50 mm molds, cured in a chamber (YH-40B) maintained at constant temperature and humidity for one day, and then demolded. Finally, the demolded specimens were further divided into three groups and cured in a water bath (HH-6, Shanghai Lichen Bangxi Instrument Technology Co., Ltd., Shanghai, China) at three different temperatures (25, 60, and 90 °C) for three days. The sample preparation process is shown in Figure 2.

### 2.3. Testing Methods

**Figure 3:**
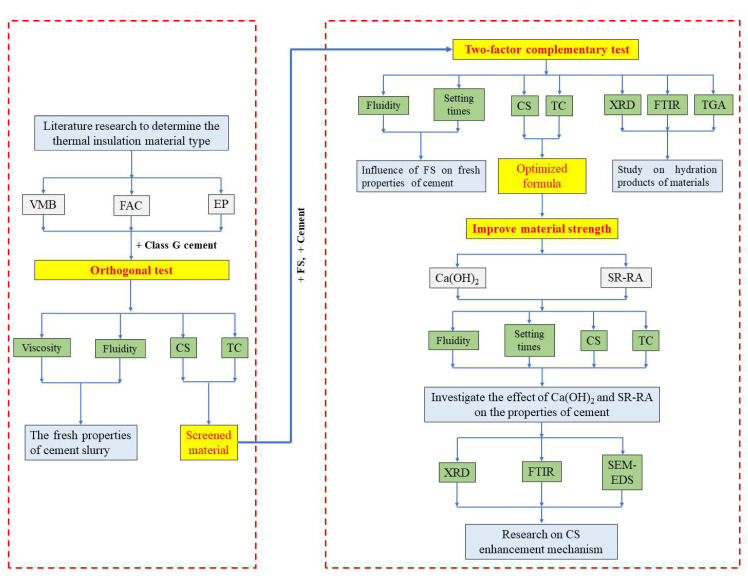
A schematic illustration of the experimental design.

In this study, the experimental steps were divided into three main parts. (1) Orthogonal test, with the evaluation criteria of a high CS and low TC, was used to screen inorganic TIMs. (2) Two-factor complementary test, in which FS was introduced into the cement with the screened material, was to obtain a lower thermal conductivity of TIC. (3) The compressive strength improvement test, based on the optimized formulation derived from step (2), was to enhance the CS of TIC with optimized TC. In the first step, the fluidity and rheological properties of cement slurries were tested at ambient temperature to study the influence of additive types and dosage on cement workability. Since FS with a large specific surface area was added, although the cement slurries in (2) and step (3) were uniform, they had poor workability; their viscosities were not measured. The setting times of cement slurries in steps (2) and step (3) were tested at 90 °C for geothermal application. XRD, FTIR, and TGA were used to investigate the hydration products of TIC in step (2). Finally, the influence mechanisms of Ca(OH)_2_ and SR-RA on cement compressive strength were studied by conducting XRD, FTIR, and SEM-EDS tests. The schematic illustration of the experimental design is shown in Figure 3.

The cement slurry density was measured using a digital density meter (YMS 0.01–7.0, Qingdao Tongchun Petroleum Instrument Co., Ltd., Qingdao, China) at ambient temperature. The fluidity was measured at ambient temperature, according to the Chinese National Standard GB/T 8077-2012 [38], and the test procedure was described in a previous study [39]. The shear stress (*τ*) of the slurries at different shear rates (*γ*) (5.11, 10.21, 170, 340, 511, and 1021 s^−1^) were tested by a six-speed rotating viscometer according to API Recommended Practice 10B-2 [40]. The plastic viscosity (*μ_p_*) and yield point (*τ*_0_) are the slope and intercept, respectively, of the graph created after fitting the shear rate against the shear stress in the Bingham law [41,42]. The setting times were measured according to the Chinese National Standard GB/T1346-2011 on a Vicat needle apparatus (Zhongkejiancai ISO, Wuxi, China) [43].

After curing or immersion, the CS of the specimens was tested immediately under ambient conditions using an electro-hydraulic servo universal testing machine (YAW-300D, Jinan Wantest Electrical Equipment Co., Ltd., Jinan, China), according to ASTM C1609/C1609M. The compressive tests were performed on 5 cm × 5 cm × 5 cm sample cubes. The other samples were immersed in anhydrous ethanol to stop hydration for 4 h, and then dried to constant weight at 105 °C in an oven (DHG-9040, Changzhou Gaode Instrument Manufacturing Co., Ltd., Changzhou, China) [44]. The dried cubes were cut into 50 × 50 × 10 mm samples for the TC test. The TC was determined according to the ISO22007-2 standard using the hot disk and transient-plate heat-source method (TPS2500S) at different temperatures. The test steps of thermal conductivity are as follows. (1) Select the appropriate hot disk sensor according to the size of the sample. (2) Sandwich the hot disk sensor between two pieces of identical samples and ensure that there is no gap between them. (3) Adjust the test parameters, the measurement time, and test power so that the TC of the sample reaches a stable value.

The pore-size distribution of the samples was measured using mercury intrusion porosimetry (MIP; AutoPore IV 9500 V1.09, Micromeritics, Norcross, GA, USA) with a contact angle of 130° and a range of 0.003–146 µm. The microstructure of the TIC was analyzed using a scanning electron microscope (SEM; Zeiss Gemini 300) with a maximum magnification of 20.00 kX, equipped with an energy dispersive X-ray spectrometer (EDS; Oxford INCA). Fourier-transform infrared (FTIR) spectroscopy (Thermo-Scientific IS5, USA) was conducted in the range of 400–4000 cm^–1^ on the powdered samples obtained by grinding the hardened samples. The cement hydration products were identified via XRD analysis using a Bruker D8-Focus diffractometer emitting nickel-filtered Cu-Kα radiation (k = 1.5406 Å) at 40 kV and 40 mA. The thermal degradation behaviors of the hydrated samples were analyzed using thermogravimetric analysis (TGA; PerkinElmer Thermal Analysis) in the range of 30–800 °C at a 10 °C/min heating rate under N_2_ gas.

## 3. Results and Discussion

### 3.1. Orthogonal Test and Analysis

The fresh properties test results of the control and cement slurries of orthogonal tests are listed in Table 9. The original test results of the shear stress (*τ*) of the samples at different shear rates (s^−1^) are listed in Table A1 of Section Appendix A. These tests were conducted at ambient temperature to investigate the influence of type and replacement level of TIM on cement workability. By comparing the plastic viscosity of sample control, OE1, OE8, and OE6, it was found that the *μ_p_* of the control first decreased and then increased when the replacement level of VMBs ranged from 10 to 30%. When compared to EP, VMBs with lower water absorption has a higher surface vitrification rate [45]. The 10% VMBs reduced the viscosity and increased the fluidity, but a high replacement level of cement with VMBs still increased the water requirement, resulting in an increase in viscosity and a decrease in fluidity. The results of OE4, OE2, and OE9 show that the *μ_p_* of the control sharply increased to 158.71 mPa·s as the 10% replacement of cement with EP and continued to increase with the increase in replacement level. Since the higher water absorption and open pores of EP particles, EP significantly increased viscosity and reduced fluidity [46,47,48]. The results of OE7, OE5, and OE3 revealed that the round shape FACs significantly decreased the viscosity and increased the fluidity. There are several reasons that FAC performed exceptionally well in terms of fluidity or workability in this research. First, the spherical shape forms a low surface area, which leads to less water requirement [49]. Second, since the W/Ss of the cement slurries were maintained at 0.5, the replacement of cement with FACs enhanced the W/C ratio. Finally, the wet spherical FACs behave similarly to ball-bear leading to the increased workability and fluidity of cement [50].

The CS and TC results for the samples in the orthogonal experiments are listed in Table 9. The factors influencing the mix design were analyzed. Range analysis was used to describe the discreteness of the data, which reflects the variation range and discreteness degree of the variable distribution: the greater the scope, the greater the degree of dispersion, and vice versa [51]. Furthermore, the greater the range (R) value, the larger the change, and the greater the impact.

The results of the range analyses of CS and TC are shown in Table 10. The R values of CS were in the order R_B_ (12.86) > R_A_ (9.94) > R_C_ (2.52). This indicates that the type of thermal insulation material (Factor B) has the greatest influence on CS, followed consecutively by the curing temperature (Factor A) and mass fraction of the thermal insulation materials (Factor C). The optimum parameter combination for CS is a curing temperature of 90 °C (third level of factor A), VMB (first level of factor B), and a 20% mass fraction (second level of factor C). The factors affecting TC followed the order R_C_ (0.11) > R_B_ (0.028) > R_A_ (0.023), indicating that the mass fraction of the thermal insulation material (Factor C) had the most significant influence on TC, followed consecutively by the type of thermal insulation material (Factor B) and curing temperature (Factor A). The optimum parameter combination for a low TC is a curing temperature of 25 °C (first level of factor A), VMB or EP (first level or third level of factor B), and a 30% mass fraction (third level of factor C).

In general, both CS and TC decreased with the incremental replacement level of these three TIMs. Since the TC of air is 0.029 W/(m·K), which is much smaller than that of the solid phase of cement-based materials [52]. A large number of pores in the TIMs reduced the heat transfer speed, resulting in the thermal insulation of porous cement-based composites [45]. Meanwhile, these pores also weakened the structure of cement, leading to lower strength. When compared with EP, VMBs with a smaller particle size has greater strength and low water absorption [53]. Therefore, the strength reduction rate of the cement with VMBs was lower than that of the cement with EP at the same replacement level. Excessive FACs content in the cement led to higher porosity due to the hollow spherical shape, affecting the mechanical behavior [54]. In addition, the weak interfacial transition zone between FACs and cement matrix was also an important reason for the decrement in CS [55].

The results of the analysis of variance are listed in Table 11, where SS is the sum of the squared deviations from the mean, d*f* is the degree of freedom, MS is the mean-square, and F is the analysis of the variance of F value, respectively. Usually, depending on the confidence interval, if the significance level is < 0.05, the impact of the factor is significant. The significance of the three factors on CS is as follows: type (significance level of 0.130) > curing temperature (significance level of 0.185) > mass fraction (significance level of 0.794). For TC, this order is mass fraction (significance level of 0.009) > type (significance level of 0.195) > curing temperature (significance level of 0.280). Only the impact of the mass fraction on TC was significant, and the impacts of the type and curing temperature were comparatively small.

The analyses of variance and orthogonal experiments results have two implications. (1) The impacts of the VMBs and EP on cement properties were similar. Since low permeability is usually required in geothermal cementing, a VMB with closed cells was selected [56]. (2) TC rapidly decreased with increasing mass fraction. As the effect of the TIM mass fraction on TC was particularly significant, it was increased and maintained at 60% in order to obtain a lower TC for the subsequent Two-factor complementary tests. FS was added to the cement with VMBs.

### 3.2. Two-Factor Complementary Test

The total mass fractions of the VMBs and FS were maintained at 60%. The FS content increased from 10% to 30%, whereas the VMB content decreased from 50% to 30%. The mix proportions of cement slurries for the Two-factor complementary test are listed in Table 12. The TC and CS of FS0V60 (i.e., the cement containing only 60% VMBs) are not displayed, as the high VMB content prevented the cement from hardening. Owing to the large specific surface area and strong water absorptivity of these two insulation materials, the W/Ss of these samples cannot be unified. The W/S ratio was adjusted to maintain the flowability of the cement slurry [16]. The results of the CS, TC, slurry density, fluidity, and setting times for the samples are listed in Table 13. The results show that the high replacement level of VMBs and FS significantly decreased the fluidity of cement. Both the initial and the final setting times of the cement slurry were shortened by incorporating 60% VMBs and FS. Furthermore, the reduction rate increased with the incremental replacement level of FS. Decreasing the setting times can be thought of as being caused by two reasons in this study. (1) The high replacement levels of these two TIMs increased the water requirement, which decreased the ratio of free water to cement and thus shortened the setting times. (2) For the cement with FS incorporation, the FS particles reacted rapidly with CH to form C–S–H gels due to their very high pozzolanic reactivity and then shortened the cement setting times [57,58,59].

The TC and CS of TIC are affected by many factors, such as the W/C ratio, density, type, and mass fraction of the additives [60]. As shown in Table 13, the TC values of FS30V30, FS20V40, and FS10V50 were lower than 0.3 W/(m·K), indicating that the increased content of TIMs contributed to the reduction in TC; and the simultaneous mixing of FS and VMBs with cement is beneficial for reducing the TC of the material. The mechanism of VMB in reducing TC has been mentioned in Section 3.1. The relatively low thermal conductivity of FS should be ascribed to its multi-nanopores network and high porosity [61]. When FS was incorporated into the cement, its remained nanopores network and high porosity also contributed to the increment in the high proportion of nanopores in the cement and then the restrained TC of the cement. In addition, when compared to crystalline phases, the non-crystalline (glassy or amorphous) C–S–H gel formations result in lower thermal conductivity values [62]. The incorporation of FS in cement promoted the C–S–H gel formations, resulting in a further decrease in TC.

The CSs of FS30V30, FS20V40, and FS10V50 all decreased by more than 85% compared with that of the control. Furthermore, both TC and CS decreased in the order Control > FS20V40 > FS30V30 > FS10V50. This CS result is consistent with Refs. [59,63], which reported that a small FS content could enhance cement strength. The FS tends to physically fill the void space between the larger particles. Owing to its high specific surface area, a small amount of FS can absorb the cement hydration products to block the capillary pores. Moreover, with time, they react chemically with calcium hydroxide to produce additional material such as pozzolanic C–S–H gels, combine some water in their products and reduce in this way the porosity of the matrix and interfacial transition zone [59,64]. However, if the incorporation of FS leads to a significantly larger W/C ratio, it will decrease the cement strength [57]. The decrement in CS might be caused by several reasons in this study. First, the W/Ss of FS30V30, FS20V40, and FS10V50 were raised from 0.5 to 2.57, 2.11, and 1.52, respectively, which is harmful to CS. Second, since both FS and VMB have high specific surface areas, such a high replacement level resulted in less cement adhered on the surface of FS agglomerations and VMBs. Third, the low mix proportion of cement also resulted in a decrease in the cement matrix of TIC, affecting the formation of hydration reactants and the pore structure, and thus led to less mechanical strength. Finally, a large number of pores in TIMs weakened the cement structure, contributing to the decrement in CS. In addition, owing to the weak bonding of the VMBs to the cement matrix, the CS also decreased with an increase in the VMB content [65].

In general, the trends in the variation of TC and CS became complicated as the W/C ratio of the samples was adjusted, and FS was crushed during preparation. In this study, the CS initially increased and then decreased with an increase in the FS content, and the changing trend of TC was similar. The reason for the same trend is that both CS and TC are generally related to densification, and the denser the material, the higher its CS and TC [47]. The increased TIM content results in decreases in CS and TC, as normally, its addition increases the W/C ratio and decreases the cement density [57,66]. However, the cement containing FS experienced a delayed workability achievement time, which is likely caused by the delay of sufficient distribution of mixing water because of the smaller particle size and higher specific surface of TIMs [67]. The extended stirring time of the dispersing FS might partially account for the TC of FS20V40 being larger than that of FS10V50 [68]. Longer durations of stirring might destroy the three-dimensional (3D) nanoporous structure of the FS [69]. By contrast, the 10% FS in the FS10V50 slurry was easier to disperse uniformly. The preserved nanoporous structures in the FS, combined with the large number of pores in the VMBs, together reduced the cement density and barrier of the heat-transfer path [70]. Consequently, although the FS content of FS20V40 was larger than that of FS10V50, the greater fragmentation led to a larger TC. However, the TC of FS30V30 was lower than that of FS20V40, indicating that despite the extended stirring time leading to the crushing of the FS, the preserved nanoporous structures of the FS, present in large quantities, still reduced the TC. Considering that the FS10V50 samples had the lowest TC of 0.1836 W/(m·K) and a moderate CS of 3.5 MPa, further research was conducted to improve the CS of FS10V50 (Section 3.6).

### 3.3. XRD

**Figure 4:**
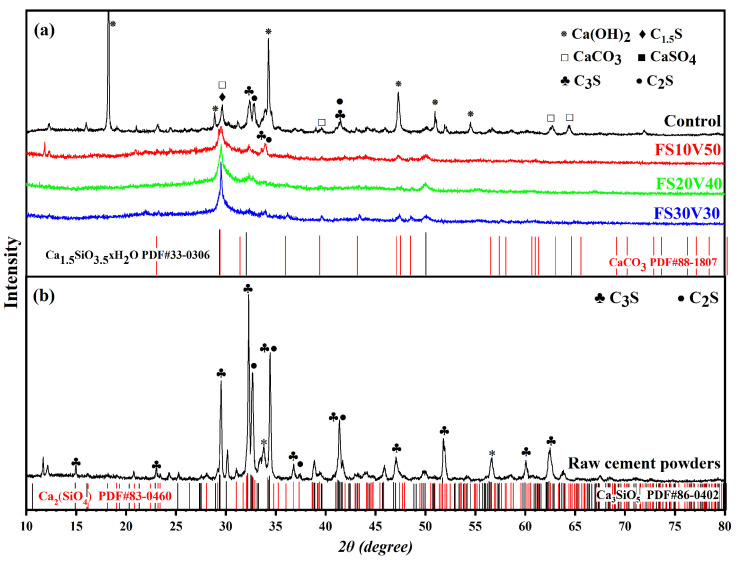
XRD patterns of (**a**) samples and (**b**) cement raw material.

The phases of the cement hydration products can be analyzed using XRD, and the relative content of each phase can be compared according to the measured diffraction intensity [71]. The XRD patterns of the samples in Section 3.2 and Class G oil-well cement powders are shown in Figure 4. The spectra of the raw cement powders revealed that they are mainly composed of alite (C_3_S) and belite (C_2_S) minerals, along with a small amount of CH formed because of moisture. The strongest diffraction peaks in the XRD pattern of the control are mainly concentrated at 18° and in the range of 30–36°. The peaks at 18.29, 34.26, 47.34, and 54.55° correspond to the crystalline phase of CH, which is an important hydration product of cement [72,73]. The peak at 29.66° was assigned to hydrated calcium silicate (C_1.5_S) and CaCO_3_. The XRD patterns of FS10V50, FS20V40, and FS30V30 were similar and relatively flat compared with that of the control, featuring few obvious peaks. The CH peaks in the patterns of FS10V50, FS20V40, and FS30V30 were not obvious, which is consistent with the results of the TGA analysis in Section 3.5, indicating that the CH content decreased with the addition of FS and VMBs [32,69]. This could be due to the pozzolanic reaction of FS consumed by CH in the cement [74]. Both the increased C–S–H gel amount owing to the pozzolanic reaction and the reduced cement density resulting from the porous structures of FS contributed to the function of FS in reducing the TC of the cement [75].

### 3.4. FT-IR Spectroscopy

**Figure 5:**
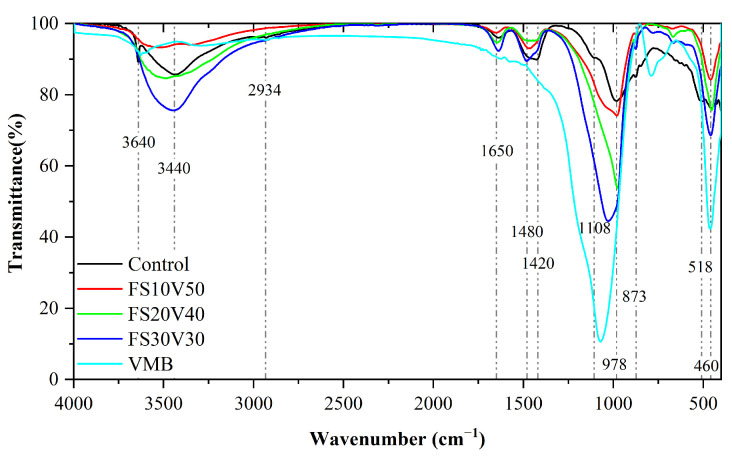
FTIR spectra of samples and VMB.

Figure 5 shows a comparison of the FT-IR spectra of the samples in the wavenumber range of 400–4000 cm^–1^. These strongest absorption bands are located at 3440.8, 1636, 1471, 1426.9, 980.6, 879.3, 667.7, and 458.3 cm^–1^, respectively. In the spectrum of the control, the sharp peak at 3640 cm^–1^ was assigned to Ca(OH)_2_, which formed during the cement hydration reaction [76]. However, this sharp peak did not appear in the other spectra, indicating that the cement hydration reaction was inhibited or that the CH was consumed [77]. This result is consistent with the absence of the diffraction peak at 18° from the XRD patterns. The absorption band at 1420 cm^–1^ corresponds to the C–H bending vibration of –CH_2_ [78]. The absorption bands at ≈1420 cm^–1^ and ≈897 cm^–1^ arise from the C–O vibration of CaCO_3_, respectively, which were generated by the carbonation of the hydration products [79]. The spectra also show bands at 1108 cm^–1^, which correspond to SO_4_^2–^ vibrations in sulfates [76].

The broad absorption band at 3200–3600 cm^–1^ corresponds to the stretching vibration of O–H, indicating the formation of C–S–H [16,76,79]. The intensity of this peak increased in the order FS30V30 > FS20V40 > FS10V50, indicating that the C–S–H content increased as the FS content increased. This is because the pozzolanic reaction of FS generates C–S–H, and the nanoporous structure of FS has a catalytic effect on the reaction [59,80]. The absorption band at ≈980.6 cm^–1^ was assigned to the asymmetric bending vibration of Si–O–Si or the vibration of the Si–OH bond in FS [16,78,81,82]. The characteristic peak of VMBs is located at 1078 cm^–1^; with increasing VMB content, this peak shifts to higher wavenumbers (i.e., FS30V30 < FS20V40 < FS10V50). The absorption band at 458.3 cm^–1^ corresponds to the bending vibration of the O–Si–O bond [16]. The highest peak intensities at 980.6 cm^–1^ and 458.3 cm^–1^ occur in the spectrum of the VMBs, followed consecutively by FS30V30, FS20V40, FS10V50, and the control, indicating that there are more Si–O bonds in VMBs and FS than in oil-well cement. In particular, FS possesses multiple silanol Si–O bonds [82].

In general, the absorption band positions of FS30V30, FS20V40, FS10V50, and the control were similar, indicating that the main chemical compositions of the FS and VMBs resembled that of the cement. The intensities of the main peaks in these spectra generally followed the order FS30V30 > FS20V40 > FS10V50 > control. Both the XRD patterns and FTIR spectra indicated the reduction in CH and the increase in C–S–H, resulting from the pozzolanic reaction of FS, which consumed CH and generated C–S–H. Since the high specific surface area of FS increases the hydrate nucleation point and shortens the ion nucleation distance, it has a certain ability to catalyze the pozzolanic reaction [57,59,63,64,83,84,85].

### 3.5. TGA

**Figure 6:**
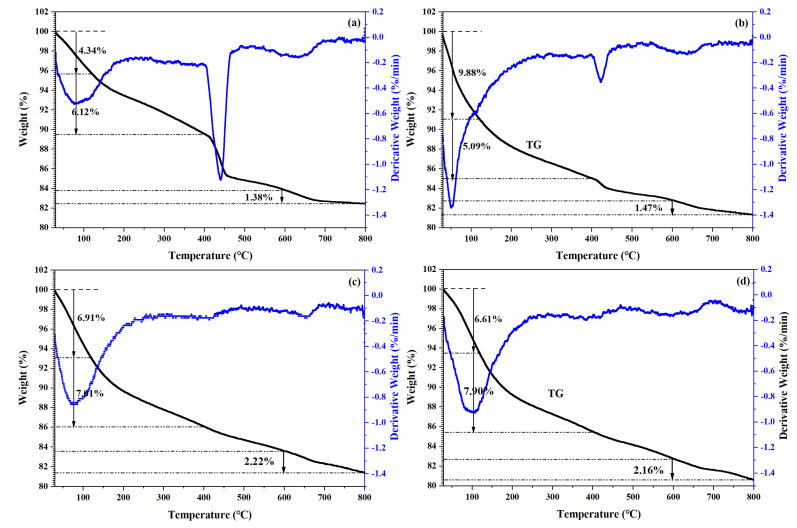
TG/DTG curves of control (**a**), FS10V50 (**b**), FS20V40 (**c**), and FS30V30 (**d**).

The expected reactions occurred in oil-well cement when subjected to a progressive temperature increase from room temperature to 800 °C in N_2_, as shown in the TG/DTG curve in Figure 6a [86]. The first weight-loss stage, from room temperature to 105 °C, was attributed to the evaporation of weakly bound water, while the second significant weight loss between 120 and 400 °C corresponds to the dehydration of C–S–H and ettringite. The third stage at 400–500 °C corresponds to the dehydration of CH. The final stage at 600–800 °C is due to the decomposition of calcium carbonate [87,88].

For the control, the weight loss in the second stage was 6.12%, which decreased to 5.09% for FS10V50 but increased to 7.01% and 7.90% for FS20V40 and FS30V30, respectively. This trend implies that the C–S–H content increased as the FS content increased. The TG/DTG curve of the control exhibited a distinct change in slope in the range of 400–500 °C, indicating the existence of CH. This change is weaker in the TG/DTG curve of FS10V50 and disappears in Figure 6c,d, indicating that the CH content decreased as the FS content increased. The TG/DTG results are consistent with the XRD and FTIR spectroscopy results. As indicated by Equation (1), because the pozzolanic reaction of FS consumes a large amount of CH and generates C–S–H, the CH content is reduced, but the C–S–H gel content increases as the FS content increases [16]:

(1) $${\mathrm{SiO}}_{2}{+\mathrm{Al}}_{2}O_{3}+2\mathrm{Ca}{\left(\mathrm{OH}\right)}_{2}{+2H}_{2}O\to \mathrm{C-S-H}+\mathrm{C-A-H}$$

### 3.6. Compressive Strength Enhancement Mechanism

#### 3.6.1. Compressive Strength

In Section 3.2, FS10V50 was chosen as the basic formula, and its CS was further improved by introducing the 10% additives SR-RA or Ca(OH)_2_. As shown in Table 14, the fluidity of FS10V50S and FS10V50C increased to 14.2 and 10.1 cm, respectively, indicating that both SR-RA and Ca(OH)_2_ have improvement effects on cement workability. Since the cement reinforcing agent generally contains a water-reducing agent, which is melamine sulfonate [89], the cement fluidity was effectively increased by adding SR-RA. The setting time results show that the shortened effect of Ca(OH)_2_ is greater than that of SR-RA at 90 °C. The CS of FS10V50 was increased to 5.85 MPa by adding 10% SR-RA but decreased by adding 10% Ca(OH)_2_. The addition of CH shortened the setting times but decreased the CS, resulting from that excessive CH addition can increase the early reaction rate but is not effective in improving the reaction rate at the later ages [90,91]. Furthermore, it is believed that excessive calcium hydroxide reduces cement strength, as excessive alkali adsorption on the surface of cement particles prevents hydration [92,93].

#### 3.6.2. FTIR

**Figure 7:**
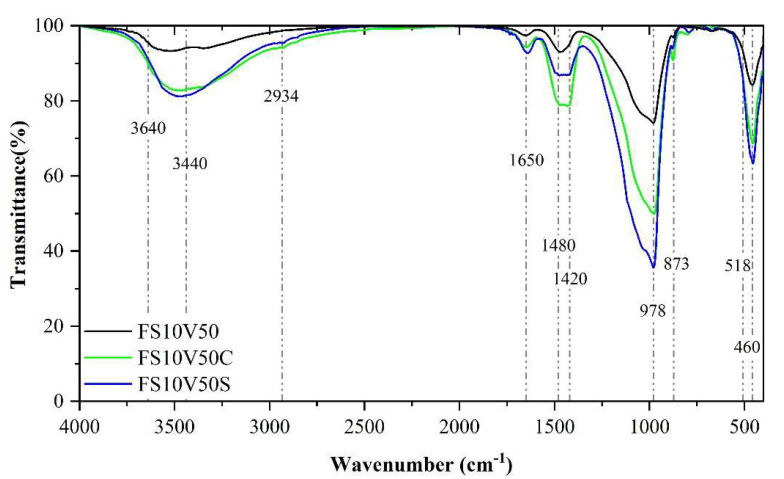
FTIR spectra of FS10V50, FS10V50S, and FS10V50C.

The FTIR spectra of FS10V50, FS10V50S, and FS10V50C are shown in Figure 7. The peak intensities of FS10V50S were higher than those of FS10V50 and FS10V50C, except for the peaks at 1433 cm^–1^ and 1474 cm^–1^, which correspond to C–O related to calcite. The increase in CaCO_3_ resulted from the carbonization of the added Ca(OH)_2_. The peaks of FS10V50S and FS10V50C in the range of 3600–3200 cm^–1^, which correspond to the stretching vibration of O–H in C–S–H, are located at higher wavenumbers than that of FS10V50. Moreover, the Si–O–Si asymmetric bending vibration of FS10V50S at 980 cm^–1^ is higher than that of FS10V50C. This phenomenon indicates that both SR-RA and calcium hydroxide promoted the pozzolanic reaction of FS and thus the formation of C–S–H. The promotional effect of SR-RA was stronger than that of calcium hydroxide, resulting in a denser Si–O–Si network structure and greater CS. The inhibitory effect of calcium hydroxide on the hydration reaction of cement led to a reduction in the CS of FS10V50C.

#### 3.6.3. MIP

**Figure 8:**
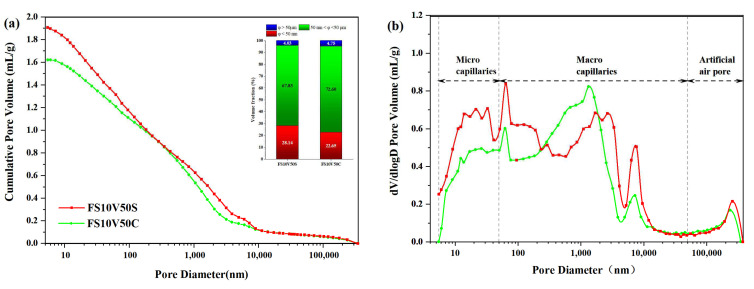
(**a**) Cumulative pore volume and (**b**) differential pore volume curves of FS10V50S and FS10V50C.

The pores of cementitious materials are typically divided into three types: micro-capillaries (φ < 50 nm), macro-capillaries (50 nm < φ < 50 μm), and artificial air pores (φ > 50 μm) [94]. The FS can reduce macroscopic capillaries and thus effectively reduce harmful pores [63]. The number of artificial air pores is related to the bonding between the FS and matrix [95]. Figure 8 shows the cumulative pore volume (a) and differential pore volume (b) curves of FS10V50S and FS10V50C. The results are summarized in Table 15. The results show that although the total pore volume of FS10V50C was smaller than that of FS10V50S, the median pore radius of FS10V50C was larger than that of FS10V50S. The volume fraction of pores > 50 nm in FS10V50C was larger than that of FS10V50S. As reported in the Refs. [96,97], cement tends to have a smaller volume fraction of pores > 50 nm with increasing age; that is, the volume fraction of pores < 50 nm in C–S–H increases as the degree of hydration increases. The smaller volume fraction of pores > 50 nm in FS10V50S compared to FS10V50C indicates that the hydration reaction degree of the TIC with SR-RA was higher than the TIC with Ca(OH)_2_. As mentioned in Table 5, there is 78.22% SiO_2_ in SR-RA, which was obtained from microspheres of granite and silica fume (Substances different from FS [98]). The silica fume has been demonstrated to have the ability to enhance the strength of the hardened cement paste by accelerating the hydration rate and increasing the C–S–H gel [99,100]. This phenomenon also explains why FS10V50C, which has lower porosity and higher density than the other two samples, has a lower CS. Excessive Ca(OH)_2_ inhibited the hydration reaction, thus weakening it.

#### 3.6.4. SEM&EDS

**Figure 9:**
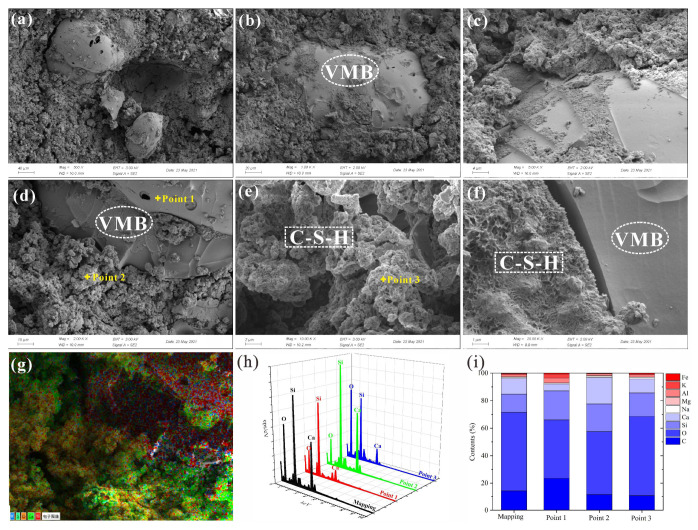
(**a**–**f**) SEM images of FS10V50S and (**g**–**i**) EDS analysis results.

**Figure 10:**
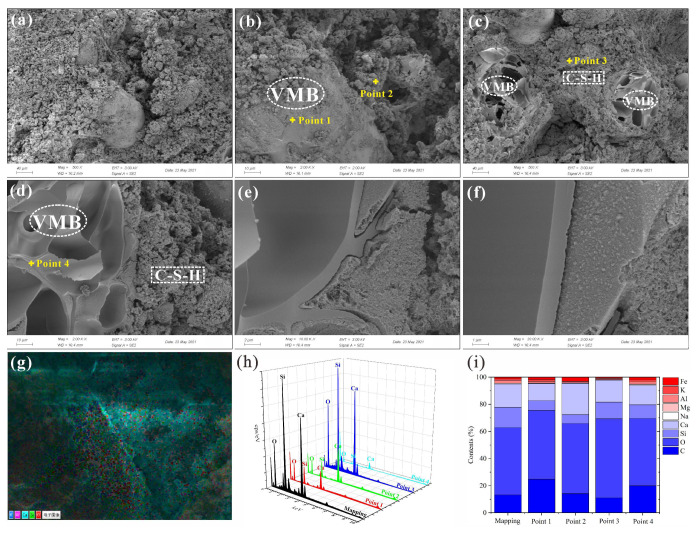
(**a**–**f**) SEM image of FS10V50C and (**g**–**i**) EDS analysis results.

The SEM images and EDS analysis results for FS10V50S and FS10V50C are shown in Figure 9 and Figure 10, respectively. Some integral VMBs, most of which are closed, are distributed in the cement. Their internal porous structure (Figure 10d) was conducive to reducing the thermal conductivity of the cement [101]. Although the inhibitory effect of calcium hydroxide on cement hydration led to a loose, non-cemented matrix with a low gel content, as C–S–H is the main product of both the hydration and pozzolanic reactions, the matrix of both FS10V50S and FS10V50C were mainly composed of C–S–H gels [16,59,63,68]. The FS powders were uniformly mixed with cement and wrapped in gel owing to their micron-sized particles and large specific surface area.

The mapping results in Figure 9i and Figure 10i show that the atomic percentage (at%) of Ca in F10V50S is smaller than that in FS10V50C owing to the addition of calcium hydroxide in the latter. The EDS spectrum of the VMB surface in F10V50S (Figure 9, point 1) also revealed a lower Ca content (4.99 at%) compared with that of FS10V50C (12.51 at%) (Figure 10, point 1). As shown in the spectra of the cement matrix near the VMBs, the C and Ca contents in F10V50S (Figure 9, point 2) are 11.53 and 19.12 at%, respectively, and those of FS10V50C (Figure 10, point 2) are 14.25 and 23.10 at%, respectively. The C and Ca contents in the cement matrix near the VMBs of F10V50S are also lower than those of F10V50S, demonstrating that some of the excess calcium hydroxide was absorbed by the VMBs and carbonated to generate CaCO_3_. This also explains the increase in CaCO_3_ in the FTIR spectrum of F10V50C in Section 3.6.2. In both samples, microcracks occurred around the VMBs owing to dry shrinkage of the cement matrix at 90 °C. This indicates that the chemical inertia of the VMB surface weakened the bond between the cement and VMBs, and explains why the material strength decreased significantly with increasing VMB content.

However, although the TC of the developed TIC decreased to 0.19 W/(m·K), with the enhanced CS of 5.85 MPa, the CS still cannot meet the requirements of geothermal cement (greater than 6.9 MPa for 24 h). Incorporating carbon nanotubes has been demonstrated to be effective in reducing shrinkage and improving the bond between cement paste and other materials [102,103]. Therefore, improving the activity of VMBs by surface modification or introducing carbon nanotubes to greatly enhance the CS of TIC should be further studied.

## 4. Conclusions

In this study, FS was introduced into cement along with a thermally-insulated additive optimized by conducting orthogonal tests. The mechanical performance of the optimized cement formula was further improved by the addition of either a cement-reinforcing agent or CH. The effects of the TIMs on the fresh properties, CS, TC, hydration products, pore-size distribution, and microstructures of cement were investigated. A TIC with a TC of 0.1905 W/(m·K) and CS of 5.85 MPa was developed for medium-low-temperature geothermal wells. The main conclusions can be summarized as follows:

(a) The order of influence of the three factors on the CS was insulation material > curing temperature > mass fraction, while the order of influence on the TC was mass fraction > type of insulation material > curing temperature. The impact of the mass fraction was particularly significant.
(b) When the total mass fractions of VMB and FS were kept at 60%, the trend reflecting the change in TC and CS became complex. The composition of FS10V50 was chosen as the basis for improving CS, as it had the lowest TC and moderate CS. As the pozzolanic reaction of the FS consumed Ca(OH)_2_ and generated C–S–H gel, the CH content decreased, but the C–S–H gel content increased as the FS content increased.
(c) The SR-RA could significantly improve the fluidity of FS10V50. The CS of FS10V50 increased to 5.85 MPa by adding 10% SR-RA but decreased by adding 10% Ca(OH)_2_. As the C–S–H gel is the main product of both the hydration and pozzolanic reactions, the matrices of both FS10V50S and FS10V50C were mainly composed of gel.
(d) The chemical inertia of the VMB surface weakened the bond between the cement and VMBs, resulting in a substantial reduction in CS. The SR-RA improved the strength by promoting cement hydration without changing the chemical inertia of the VMB surface.

## Figures and Tables

**Table 1:** Symbols and mathematical notations.

TIC	Thermal insulation cement	FAC	Fly-ash cenospheres
FS	Fumed silica	EP	Expanded perlite
TC	Thermal conductivity	TIM	Thermally insulated materials
CS	Compressive strength	SR-RA	Reinforcing agent No. SR
RA	Reinforcing agent	W/C	Water to cement ratio
CH	Calcium hydroxide	W/S	Water to solid ratio
VMB	Vitrified microbubble	*τ*	Shear stress
*μ_p_*	Plastic viscosity	*γ*	Shear rate
*τ*_0_	Yield point	R	Range
R_A_	Range of factor A	R_B_	Range of factor B
R_C_	Range of factor C	d*f*	Degree of freedom
MS	Mean square	F	Statistic inspection value
SS	Sum of the squared deviations from the mean	φ	Pore size
at%	Atomic percentage	C–S–H	Calcium Silicate Hydrate

**Table 2:** Chemical composition and physical properties of Class G oil-well cement.

C_3_S (%)	2C_3_A + C_4_AF (%)	C_3_A (%)	Loss on Ignition (%)	W/C = 0.5, 15–30 min Slurry Consistency (BC)	52 °C, 35.6 MPa, Thickening Time (min)
54.2	18.4	1.9	1.35	18.6	103

**Table 3:** SR-RA detection index.

Silica (%)	Chloride Ion Content (%)	PH Value (%)	Total Alkali Content (%)	Water Content (%)
65.88	0.26	9.35	5.45	0.75

**Table 4:** Physical properties of hydrophilic FS.

Bulk Density (kg/m^3^)	Particle Size (mm)	Porosity (%)	Specific Surface Area (m^2^/g)	Pore Size (nm)	TC (W/(m·K))
4.01	0.1–0.5	>90	800–1000	20–40	0.018

**Table 5:** Oxide composition of raw materials.

Oxide	Al_2_O_3_	CaO	SiO_2_	SO_3_	Fe_2_O_3_	Na_2_O	K_2_O	TiO_2_	MnO	MgO
Oil well cement	3.15	64.77	19.52	3.21	5.11	0.37	0.69	0.33	0.04	2.23
VMB	11.92	1.29	75.81	0.02	0.98	3.21	5.87	0.10	0.07	0.34
FAC	23.39	3.63	59.21	1.28	4.80	1.61	3.09	1.10	0.05	1.31
EP	13.54	1.58	75.84	0.05	0.67	2.89	4.84	0.09	0.04	0.37
SR-RA	1.56	3.58	78.22	9.96	0.90	3.77	0.74	0.09	0.02	0.73

**Table 6:** Three factors and their corresponding levels.

Factors	Curing Temperature (°C)	Type of Thermal Insulation Material	Mass Fraction of Insulation Materials (wt%)
Level 1	25	VMB	10
Level 2	60	EP	20
Level 3	90	FAC	30

**Table 7:** Orthogonal experiment table.

No.	Factors			
	Curing and Testing Temperature (°C)	Type of TIM	Mass Fraction of Insulation Materials (wt%)	Blank Column
	(Factor A)	(Factor B)	(Factor C)	
OE1	25 (1)	VMB (1)	10 (1)	1
OE2	25 (1)	EP (2)	20 (2)	2
OE3	25 (1)	FAC (3)	30 (3)	3
OE4	60 (2)	EP (2)	10 (1)	3
OE5	60 (2)	FAC (3)	20 (2)	1
OE6	60 (2)	VMB (1)	30 (3)	2
OE7	90 (3)	FAC (3)	10 (1)	2
OE8	90 (3)	VMB (1)	20 (2)	3
OE9	90 (3)	EP (2)	30 (3)	1

**Table 8:** Mix proportions of cement slurries for orthogonal test.

No.	Cement (g)	VMB (g)	EP (g)	FAC (g)	W/S Ratio
OE1	90	10	0	0	0.50
OE2	80	0	20	0	0.50
OE3	70	0	0	30	0.50
OE4	90	0	10	0	0.50
OE5	80	0	0	20	0.50
OE6	70	0	30	0	0.50
OE7	90	0	0	10	0.50
OE8	80	20	0	0	0.50
OE9	70	0	30	0	0.50

**Table 9:** Results of the orthogonal tests.

No.	Factor A	Factor B	Factor C	Plastic Viscosity (mPa·s)	Yield Stress (Pa)	Fluidity (cm)	Density (g/cm^3^)	CS (MPa)	TC (W/(m·K))
control	/	/	/	110.68	39.35	16.2	1.84	/	/
OE1	25 (1)	VMB (1)	10 (1)	66.15	31.81	17.5	1.61	17.47	0.6622
OE2	25 (1)	EP (2)	20 (2)	160.83	36.14	10.3	1.52	9.57	0.5443
OE3	25 (1)	FAC (3)	30 (3)	45.23	27.06	16.5	1.52	9.9	0.4972
OE4	60 (2)	EP (2)	10 (1)	158.71	41.17	12.7	1.63	18.25	0.6686
OE5	60 (2)	FAC (3)	20 (2)	44.89	27.94	17.3	1.59	16.83	0.5371
OE6	60 (2)	VMB (1)	30 (3)	158.62	31.48	13.5	1.38	28.94	0.5435
OE7	90 (3)	FAC (3)	10 (1)	36.57	32.16	18.2	1.72	21.69	0.6699
OE8	90 (3)	VMB (1)	20 (2)	108.33	24.15	15.5	1.48	32.55	0.5853
OE9	90 (3)	EP (2)	30 (3)	170.17	33.69	8.5	1.43	12.53	0.5176

**Table 10:** Range analysis of CS and TC.

Performance Index	Source of Range	K_i1_	K_i2_	K_i3_	k_i1_	k_i2_	k_i3_	R_i_
CS	(Factor A)	36.93	64.01	66.76	12.31	21.34	22.25	9.94
	(Factor B)	78.95	40.34	48.41	26.32	13.45	16.14	12.86
	(Factor C)	57.39	58.94	51.37	19.13	19.65	17.12	2.52
TC	(Factor A)	1.70	1.74	1.77	0.57	0.58	0.59	0.023
	(Factor B)	1.70	1.73	1.70	0.57	0.58	0.57	0.028
	(Factor C)	2.00	1.66	1.55	0.67	0.55	0.52	0.11

**Table 11:** Analysis of variance.

Performance Index	Source of Variation	SS	d*f*	MS	F	Level of Significance
CS	Factor (A)	181.191	2	90.595	4.391	0.185 (non-significant)
	Factor (B)	276.505	2	138.253	6.701	0.130 (non-significant)
	Factor (C)	10.701	2	5.351	0.259	0.794 (non-significant)
	Error	41.265	2	20.632	/	/
	Total	3635.590	8	/	/	/
	R^2^ = 0.919 (Adjusted R^2^ = 0.676)					
TC	Factor (A)	0.001	2	0.000	2.572	0.280 (non-significant)
	Factor (B)	0.001	2	0.001	4.130	0.195 (non-significant)
	Factor (C)	0.035	2	0.018	110.855	0.009 (significant)
	Error	0.000	2	0.000	/	/
	Total	3.072	8	/	/	/
	R^2^ = 0.992 (Adjusted R^2^ = 0.966)					

**Table 12:** Mix proportions of cement slurries for Two-factor complementary test.

NO.	Cement	FS	VMB	W/S Ratio	Curing and Test Temperature (°C)
control	100	0	0	0.50	90
FS30V30	40	30	30	2.57	90
FS20V40	40	20	30	2.11	90
FS10V50	40	10	50	1.52	90

**Table 13:** Complementary test results of VMBs and FS.

No.	CS (MPa)	TC (W/(m·K))	Slurry Density (g/cm^3^)	Fluidity (cm)	Initial Setting Time (min)	Final Setting Time (min)
control	34.7	0.7265	1.84	16.2	147	185
FS30V30	2.03	0.2373	1.10	6.0	40	49
FS20V40	5.40	0.2906	1.08	6.0	45	80
FS10V50	3.50	0.1836	1.01	8.0	111	128

**Table 14:** Test results of FS10V50 with additives.

No.	Temperature (°C)	Additive Type	Dosage (%)	CS (MPa)	TC (W/(m·K))	Density (g/cm^3^)	Fluidity (cm)	Initial Setting Time (min)	Final Setting Time (min)
FS10V50	90	/	0	3.50	0.1836	1.04	8.0	111	128
FS10V50S	90	SR-RA	10	5.85	0.1905	1.00	14.2	89	110
FS10V50C	90	CH	10	1.76	0.2247	1.07	10.1	65	73

**Table 15:** MIP analysis results.

No.	Porosity (%)	Bulk Density (g/mL)	Median Pore Radius (V/nm)	Median Pore Radius (A/nm)	Total Pore Volume (mL/g)	Total Pore Area (m^2^/g)
FS10V50C	75.8	0.46	412.3	16.9	1.61	100.0
FS10V50S	78.4	0.41	224.2	14.6	1.90	154.5

## Data Availability

The data presented in this study are available from the corresponding authors upon reasonable request.

## Appendix A

**Table A1:** Raw data of shear stress (Pa) at different shear rates (s^−1^) of cement slurries.

NO.	Shear Stress (Pa) at Different Shear Rate (s^−1^)					
	1021.8	510.9	340.6	170.3	10.22	5.11
Control	140.53	112.42	84.83	65.41	33.22	27.59
OE1	94.54	68.99	61.32	48.55	30.66	23.00
OE2	/	107.31	104.76	68.99	40.88	25.55
OE3	66.43	58.77	48.55	38.33	25.55	17.89
OE4	/	109.87	107.31	81.76	38.33	33.22
OE5	68.99	53.66	51.10	40.88	27.59	17.89
OE6	0.00	107.31	91.98	61.32	33.22	28.11
OE7	66.43	53.66	48.55	40.88	33.22	25.55
OE8	137.97	71.54	61.32	47.01	28.11	21.97
OE9	/	109.87	104.76	68.99	35.77	25.55
